# METABOLIC DYSFUNCTION-ASSOCIATED STEATOTIC LIVER DISEASE — ASSESSMENT OF PATIENTS WITH OBESITY AND METABOLIC SYNDROME — GUIDELINE FROM THE BRAZILIAN SOCIETY OF BARIATRIC AND METABOLIC SURGERY

**DOI:** 10.1590/0102-6720202400028e1821

**Published:** 2024-09-02

**Authors:** Leonardo HALAMY PEREIRA, Fernando de BARROS, Thais Guaraná de ANDRADE, Alvaro Albano de OLIVEIRA, Cristiane Alves Villela NOGUEIRA, Antonio Carlos VALEZI

**Affiliations:** 1 Universidade Federal Fluminense, Department of Specialized and General Surgery – Niterói (RJ), Brazil;; 2 Universidade Federal Fluminense, Department of Clinical Medicine – Niterói (RJ), Brazil;; 3 Santa Casa de Misericórdia de Itabuna, Bariatric Surgery Service – Itabuna (BA), Brazil;; 4 Universidade Federal do Rio de Janeiro, Department of Clinical Medicine – Rio de Janeiro (RJ), Brazil;; 5 Universidade Estadual de Londrina, Department of Surgery – Londrina (PR), Brazil.

**Keywords:** Bariatric surgery, Fatty liver, Metabolic syndrome, Obesity, Cirurgia bariátrica, Fígado gorduroso, Síndrome metabólica, Obesidade

## Abstract

**BACKGROUND:**

Metabolic dysfunction-associated steatotic liver disease (MASLD) is the most prevalent chronic liver disease in the world and was recently renamed to emphasize its metabolic component.

**AIMS:**

This article seeks to fill the gap in specific guidelines for patients with obesity and MASLD who will undergo bariatric surgery.

**METHODS:**

A systematic search for guidelines was carried out on PubMed and Embase platforms.

**RESULTS:**

A total of 544 articles were found, of which 11 were selected according to inclusion and exclusion criteria. All 11 guidelines are from clinical societies; therefore, they do not include some necessary interpretations for bariatric patients.

**CONCLUSIONS:**

We recommend that every patient undergoing bariatric and metabolic surgery be screened initially with the Fibrosis-4 (FIB-4) score, followed by transient hepatic elastography (vibration-controlled transient elastography, VCTE), especially for those with FIB-4>1.3. However, interpreting VCTE results in obese patients requires further studies to define the actual cutoff values. Enhanced Liver Fibrosis^®^ shows promise but its availability is limited. The indication for liver biopsy during surgery needs to be individualized but it is recommended for those with changes in FIB-4 and/or VCTE. Family screening is recommended for relatives of young patients with already advanced fibrosis. Liver transplantation is an option for patients with advanced MASLD but the optimal timing for bariatric surgery with transplantation is still unclear. Regular follow-up and VCTE examination are recommended to monitor disease progression after surgery.

## RESUMO

**RACIONAL:**

A doença hepática esteatótica associada à disfunção metabólica (MASLD) é a doença hepática crônica mais prevalente no mundo e foi recentemente renomeada para enfatizar seu componente metabólico.

**OBJETIVOS:**

Este artigo busca preencher a lacuna de diretrizes específicas para pacientes portadores de obesidade e MASLD que irão ser submetidos à cirurgia bariátrica.

**MÉTODOS:**

Foi realizado uma busca sistemática por diretrizes nas plataformas PubMed e Embase.

**RESULTADOS:**

Foram encontrados 544 artigos, dos quais 11 foram selecionados conforme critérios de inclusão/exclusão. Todas as 11 diretrizes são de sociedades clínicas, portanto, não contemplam algumas interpretações necessárias para o paciente bariátrico.

**CONCLUSÕES:**

Recomendamos que seja feito o rastreio de todo paciente que será submetido a cirurgia bariátrica e metabólica inicialmente com o *score* Fibrosis-4 (FIB-4), seguido da elastografia hepática transitória *(vibration-controlled transient elastography — VCTE)*, principalmente para aqueles com FIB-4>1,3. No entanto, a interpretação dos resultados do VCTE em pacientes obesos necessita de mais estudos para delimitar os reais valores de cortes. O *Enhanced Liver Fibrosis* ^®^ mostra-se promissor, mas sua disponibilidade é limitada. A indicação da biópsia hepática durante a cirurgia, precisa ser individualizada, porém é recomendada para aqueles com alteração do FIB-4 e/ou da VCTE. O rastreio familiar é recomendado para parentes de pacientes jovens já com fibrose avançada. O transplante hepático é uma opção para pacientes com MASLD avançada, mas o momento ideal para a cirurgia bariátrica em relação ao transplante ainda não está claro. O acompanhamento regular e o exame VCTE são recomendados para monitorar a progressão da doença após a cirurgia.

## INTRODUCTION

Metabolic dysfunction-associated steatotic disease (MASLD) has an estimated global prevalence of 38.7%, making it the most common chronic liver disease worldwide^6^. Its prevalence is estimated at 69.9% in the overweight population and 75.3% in the obese^25^. The significant increase in obesity in recent decades has been accompanied by advanced liver disease, including advanced fibrosis, cirrhosis, liver cancer, and an increase in the number of transplants due to MASLD^14,15^.

In 2023, a multi-society consensus was published, conducted by the American Association for the Study of Liver Diseases (AASLD) and the European Association for the Study of the Liver (EASL), in collaboration with the Asociación Latinoamericana para el Estudio del Hígado (ALEH), which introduced the new nomenclature, replacing the term non-alcoholic fatty liver disease (NAFLD) with MASLD^13,26^. According to the opinion of the majority of specialists consulted at the meeting, the use of the term “metabolic” instead of “fatty” and “non-alcoholic” would help healthcare professionals better explain and understand the disease and avoid stigmatizing terms. Thus, patients diagnosed with hepatic steatosis, who present any evidence of metabolic dysfunction, are diagnosed with MASLD provided there are no other identifiable origins for chronic liver disease^28^.

The important progress in addressing this disease will enable bariatric surgeons and hepatologists to identify, even in the preoperative phase of bariatric and metabolic surgery (BMS), patients at high risk of advanced liver disease and plan the best approach (technique and the need for liver biopsy for better staging of the disease)^31^. There is scientific evidence that BMS is the primary and best treatment for MASLD in patients with obesity, showing improvement in steatosis and progressive regression of fibrosis in the long term, likely associated with weight loss^20,33^.

However, it is worth noting that all recently published studies and guidelines were based on population studies (including obese and non-obese individuals) focused on patients with chronic liver disease who will not necessarily undergo bariatric surgery^26^. To date, there are no studies or guidelines specifically focused on obese individuals who will undergo BMS. Considering this scenario, the Brazilian Society of Bariatric and Metabolic Surgery discusses some important care points and proposes a guideline to assist the bariatric surgeon in approaching obese patients with a potential risk for associated MASLD.

## METHODS

A systematic search for guidelines was conducted on PubMed and Embase databases that included analyses and/or recommendations directed at the obese population. There were no restrictions regarding publication date or language. The search terms used were: (“non-alcoholic fatty liver disease”[Mesh] OR “metabolic dysfunction fatty liver disease” OR “MAFLD” OR “metabolic dysfunction associated steatotic liver disease” OR “MASLD”) AND (“obesity, abdominal”[Mesh] OR “obesity”[Mesh] OR “abdominal obesity metabolic syndrome” [Supplementary Concept] OR “metabolic syndrome”[Mesh] OR “body mass index”[Mesh]) AND (“guideline adherence”[Mesh] OR “guideline” [Publication Type] OR “Guidelines as Topic”[Mesh] OR “guideline”[All Fields])” on PubMed tool and (‘nonalcoholic fatty liver’/exp OR ‘nonalcoholic fatty liver’ OR MASLD OR ‘metabolic dysfunction associated steatotic liver disease’/exp OR ‘metabolic dysfunction associated steatotic liver disease’) AND (‘abdominal obesity’/exp OR ‘abdominal obesity’ OR ‘morbid obesity’/exp OR ‘morbid obesity’ OR ‘metabolic syndrome x’/exp OR ‘metabolic syndrome x’ OR ‘body mass’/exp OR ‘body mass’) AND (‘protocol compliance’ or ‘practice guideline’) on Embase tool.

We searched for studies on screening, diagnosis, treatment, and follow-up for MASLD aimed at the obese population. Studies that were not guidelines, guideline review articles, publications not belonging to associations or scientific societies, studies restricted to the pediatric population, studies restricted to underweight or malnourished populations, and guidelines for liver diseases other than MASLD were excluded (Table 1).

**Table 1:** Included articles.

Guidelines	Year
European Association for the Study of the Liver (EASL), European Association for the Study of Diabetes (EASD), and European Association for the Study of Obesity (EASO)^12^	2016
National Institute for Health and Care Excellence (NICE)^22^	2016
Italian Association for the Study of the Liver (AISF)^17^	2017
Asia-Pacific Working Party on Non-alcoholic Fatty Liver Disease (APASL)^8,34^	2017
Association for the Study of Liver Diseases (AASLD)^5^	2018
Italian Association for the Study of the Liver (AISF), Italian Society of Diabetology (SID) and Italian Society of Obesity (SIO)^3^	2021
European Association for the Study of the Liver (EASL) clinical practice guidelines: non-invasive liver tests for evaluation of liver disease severity and prognosis^13^	2021
American Association of Clinical Endocrinology (AACE)^9^	2022
Brazilian Society of Endocrinology and Metabolism (SBEM), Brazilian Society of Hepatology (SBH), and Brazilian Association for the Study of Obesity and Metabolic Syndrome – (ABESO)^21^	2023
Association for the Study of Liver Diseases (AASLD)^27^	2023
Brazilian Diabetes Society (BDS)^16^	2024

## RESULTS

**Figure 1:**
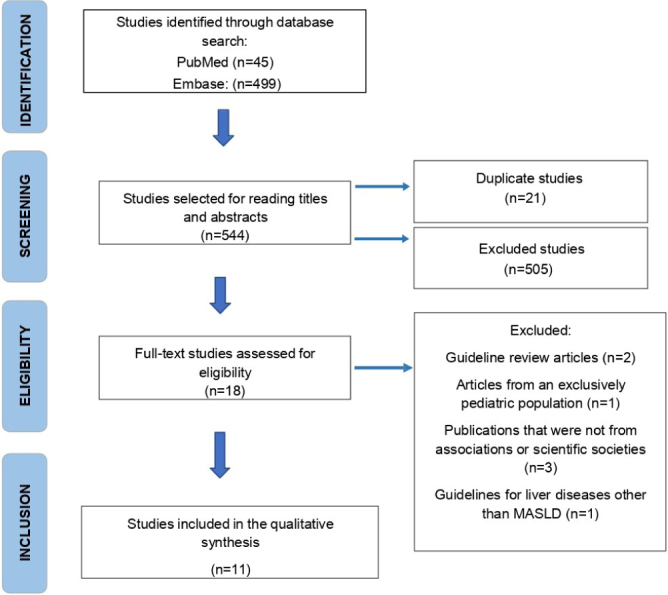
Adapted Preferred Reporting Items for Systematic reviews and Meta-Analyses (Prisma) flowchart^23^.

A total of 544 articles were identified in the described databases. After applying the inclusion and exclusion criteria, 11 guidelines remained for discussion (Figure 1)^23^. Three articles are from American societies; two from Brazilian; two from Italian; one from British; two from grouped European; and one from Asian and Pacific society. Six are publications from the last five years, and none are from surgical societies.

MASLD: metabolic dysfunction-associated steatotic liver disease.

## DISCUSSION

MASLD is closely associated with obesity and metabolic syndrome. BMS is proven to be the best treatment for the disease in the medium and long term. Clinicians, gastroenterologists, hepatologists, and bariatric surgeons should consider screening for MASLD in all patients with obesity and/or metabolic syndrome, particularly those preparing for BMS. However, the literature lacks a guideline for the diagnostic approach, management, treatment (surgical), and follow-up exclusively for this population.

### Recommendation for screening all adults with obesity and/or metabolic syndrome for the risk of advanced fibrosis associated with MASLD using the Fibrosis-4 index.

As suggested by most societies, due to ease, cost, and practicality, fibrosis screening should begin with the calculation of the Fibrosis-4 (FIB-4) score^29^. The index is determined by the mathematical formula that divides the product of age and aspartate aminotransferase (AST) by the product of platelet count and the square root of alanine aminotransferase (ALT). Various websites, platforms, and some laboratories offer this calculation automatically. The score has an area under the receiver operating characteristic (AUROC) of 0.801 for detecting advanced fibrosis in people with obesity^24^. The American Association of Clinical Endocrinology (AACE)^9^ also highlights that the FIB-4 can stratify future morbidity and mortality from liver disease. Most guidelines, although not exclusively designed for obese individuals, consider values up to 1.3 as non-advanced fibrosis. With this cutoff, there is a sensitivity of 84.4% and a specificity of 68.5% in the general population for identifying advanced fibrosis^30^. For patients with values greater than 2.67, it is understood that they are at high risk of advanced fibrosis and thus require more in-depth investigation. The Italian Association for the Study of the Liver (AISF)^17^ uses a combination of FIB-4 and NAFLD fibrosis score (NFS) for screening, with the latter having a cutoff value of 0.1455. We strongly suggest that all patients with obesity and/or metabolic syndrome with an FIB-4 greater than 1.3 continue screening for active hepatic fibrosis via another non-invasive method. For patients with values above 2.67, we recommend referral and evaluation by a hepatologist for appropriate staging (Child-Pugh score [CHILD] and Model for End-Stage Liver Disease [MELD]) and follow-up^24^.

### Recommendation for vibration-controlled transient elastography

VCTE is the most validated non-invasive technology for liver fibrosis stratification according to current guidelines^1^. The risk classification for advanced fibrosis in nearly all guidelines is based on the following values: low-risk group (VCTE<8.0 kPa); indeterminate-risk group (VCTE=8.0 to 12 kPa); and high-risk group (VCTE>12.0 kPa). However, it is important to note that there is still no consensus regarding these cutoff points in patients with obesity. In clinical practice, some discrepancies have been observed between VCTE readings and histopathological findings from biopsies performed during bariatric surgery. One possible hypothesis is that cirrhotic livers resulting from, for example, viral hepatitis are rigid. In contrast, cirrhotic livers associated with MASLD may be less hard due in part to some fat accumulation, which could lead to misleading VCTE readings, as VCTE primarily measures liver stiffness. Thus, the cutoff values might be higher in patients with obesity. More validation studies correlating biopsies and VCTE in the obese population are needed to confirm the best cutoff points for fibrosis stratification in this population.

Magnetic resonance elastography could be an alternative for obese patients in whom VCTE with the XL probe is not feasible. Alongside VCTE, the controlled attenuation parameter (CAP) score can be measured using elastography equipment. As defined by AISF^17^, this test is a good non-invasive tool, particularly for post-treatment steatosis follow-up. However, EASL, European Association for the Study of Diabetes (EASD), and European Association for the Study of Obesity (EASO)^12^ highlight the limitation in discriminating histological grades of steatosis by CAP. A meta-analysis cited by the Brazilian Association for the Study of Obesity and Metabolic Syndrome (ABESO), Brazilian Society of Hepatology (SBH), and Brazilian Society of Endocrinology and Metabolism (SBEM)^21^ indicated from 61 studies that the AUROC of CAP in the obese population is 0.88 for the diagnosis of steatosis ≥S1^4^. We therefore recommend that CAP be considered whenever there is doubt about the diagnosis of steatosis, given that the CAP score can be measured alongside VCTE. We consider CAP a useful tool for monitoring the improvement or even resolution of steatosis after BMS.

**Figure 2:**
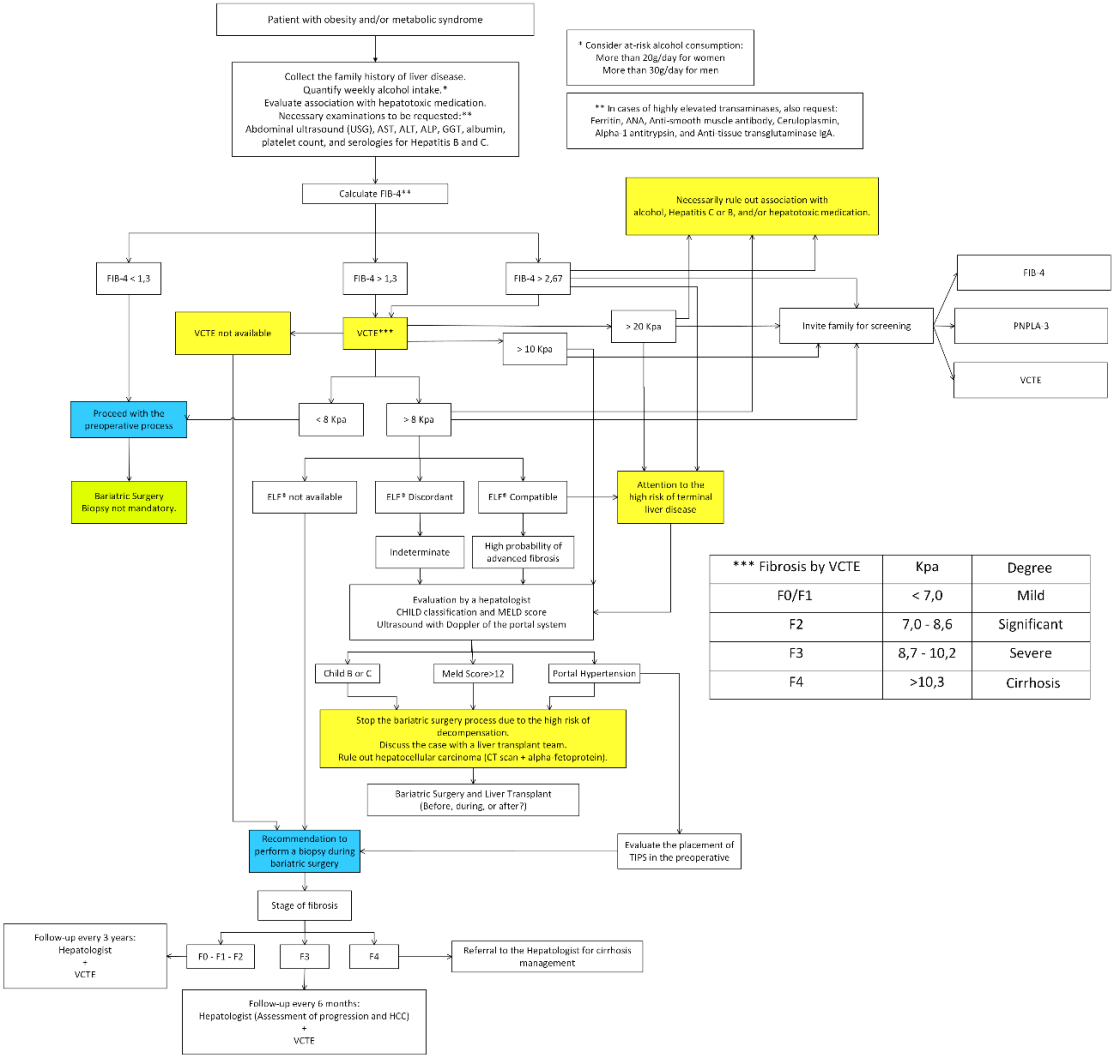
Flowchart for patients with obesity and metabolic syndrome undergoing bariatric and metabolic surgery.

FIB-4: Fibrosis-4; VCTE: vibration controlled transient elastography; ELF: enhanced liver fibrosis; PNPLA-3: patatin-like phospholipase domain-containing protein 3; CHILD: Child Pugh classification; MELD: Model for end-stage liver disease; AST: aspartate aminotransferase; ALT: alanine aminotransferase; ALP: alkaline phosphatase; GGT: gamma-glutamyl transferase; ANA: antinuclear antibody; IgA: immunoglobulin A antibody; ICT: intraoperative computed tomography; TIPS: transjugular intrahepatic portosystemic shunt; HCC: hepatocellular carcinoma.

The initial screening under the ABESO, SBH, and SBEM^21^ guidelines should be conducted through an imaging method — VCTE, ultrasonography, or magnetic resonance imaging. This can be understood as a protocol for the general population with overweight, initially seeking hepatic steatosis, whose prevalence is lower compared to the obese population. We recommend that all patients with obesity and/or metabolic syndrome with an altered FIB-4 be evaluated with VCTE if possible, as most patients with obesity already have some degree of steatosis and a smaller proportion have fibrosis. For patients who were screened and with FIB-4 greater than 1.3, evaluation with VCTE should also be recommended. If the VCTE reading is above 8 kPa, referral for evaluation by a hepatologist should always be considered. Centers without VCTE may use other serum scores for fibrosis stratification or other ultrasound elastography equipment. If no imaging test is possible, a biopsy during bariatric surgery should be considered if the FIB-4 is above 1.3 (Figure 2).

### Recommendation for the use of Enhanced Liver Fibrosis^®^ for diagnosis of liver fibrosis

This non-invasive test for liver fibrosis evaluation derives from the combination of plasma levels of three matrix renewal proteins. The ELF^®^ test has an AUROC of 0.90 for detecting advanced fibrosis in the general population^2^. The British National Institute for Health and Care Excellence (NICE)^22^ refers to ELF^®^ as having the best cost-effectiveness ratio in identifying patients with advanced stages of fibrosis in the general population. The guidelines from AACE⁹ and ABESO, SBH, and SBEM^21^ position the test as an alternative to VCTE in fibrosis stratification. A meta-analysis of 16 studies showed that ELF^®^ values above 7.70 had a negative predictive value of 0.99 for identifying advanced fibrosis^32^. AACE^9^ classifies the following groups for the risk of advanced fibrosis: low-risk when ELF^®^<7.7; indeterminate risk for ELF^®^ between 7.7 and 9.8; and high-risk for those with ELF^®^>9.8. According to NICE’s protocol^22^, screening starts with ELF^®^, considering a higher cutoff value for advanced fibrosis > 10.51. Interestingly, the guideline does not include any imaging tests for follow-up, thus considering ELF^®^ as the sole diagnostic method due to its sufficient sensitivity for identifying advanced fibrosis. We understand that ELF^®^ may not yet be a reality in all centers; therefore, despite its accuracy, it should be reserved as an alternative or a confirmation of a positive VCTE result^18^.

### Recommendation for liver biopsy

All guidelines agree that liver biopsy is the gold standard for diagnosing MASLD. However, considering that it is an invasive procedure, subject to interpretation errors, and difficult to apply to the entire obese population, percutaneous biopsy is not routinely recommended for diagnosis or as a screening method. We understand that since our patients are in the preoperative phase for BMS, the biopsy, when indicated, should be performed during the surgical procedure. AASLD^5,27^ recommends biopsy in patients with suspected MASLD associated with consistently elevated serum ferritin levels or increased iron saturation. In its most recent guideline, biopsy should be considered in case of uncertain diagnosis, such as may occur with discordant or indeterminate results from non-invasive tests^27^. AACE^9^ suggests routine biopsy during bariatric surgery due to the possibility of identifying other liver diseases. We believe that indiscriminate liver biopsy for all patients undergoing bariatric surgery is not appropriate, as the extremely high volume of surgeries performed worldwide each year would result in an increased absolute number of patients with complications such as bleeding, hematoma, bilioma, biliary fistula, etc., despite the low complication rate of liver biopsy^35^. We recommend that patients with FIB-4 and/or VCTE and/or ELF^®^ values suggestive of advanced fibrosis should undergo liver biopsy during bariatric surgery. If VCTE or ELF^®^ cannot be performed, we also recommend biopsy during bariatric surgery, especially for FIB-4>2.67 or in patients whose liver appears suspicious macroscopically.

### Recommendation for family screening (first-degree relatives) in case of patients with obesity and metabolic syndrome with advanced fibrosis

AASLD^27^ notes that differences in the presentation of MASLD can also be explained by different ethnicities and are associated with variations in the gene encoding patatin-like phospholipase domain-containing protein 3 (PNPLA-3)^11^. However, routine clinical practice currently does not recommend testing for PNPLA-3 variants, even though their association with advanced fibrosis in MASLD patients is proven. Additionally, the guideline highlights that certain genetic polymorphisms, such as PNPLA-3, are also associated with a higher susceptibility to hepatocellular carcinoma (HCC). AISF^17^ suggests that high-impact prospective studies should investigate the use of genetic risk profiles in reference centers for individual risk stratification of MASLD-HCC as well as for stratifying the risk of progression and sub-phenotyping of MASLD.

Despite the genetic variability of the disease, there is sufficient evidence to support family screening for first-degree relatives, especially for individuals with obesity and metabolic syndrome at high risk of advanced fibrosis: FIB-4>2.67; VCTE>8 KPa; ELF^®^>9.8. The active search for relatives at risk for the severe form of the disease is justified mainly as an important public health measure that can prevent the progression of patients with incipient MASLD to cirrhosis, HCC, and liver transplantation. Young patients with advanced liver disease should also have their families investigated due to the higher risk of disease progression. This measure is also justified by the lower positive predictive value of FIB-4 in this age group^24^. In the future, determining the PNPLA-3 genotype may become a useful tool for risk stratification of relatives, allowing for earlier intervention. Currently, access to PNPLA-3 testing is not a reality in most hepatology or BMS centers. However, in the future, this could have an impact on disease screening with the aim of early diagnosis (Figure 2).

### Recommendation for surgical treatment of metabolic dysfunction-associated steatotic liver disease

BMS is an effective treatment for the remission of MASLD and liver fibrosis in the medium and long term as well as for optimizing cardiometabolic health in patients with obesity and metabolic syndrome^33,35^. APASL^8,34^ emphasizes careful patient selection for bariatric surgery, highlighting its potential to improve MASLD histology and reduce long-term mortality, especially in patients with class II obesity. AASLD^5^ and AISF^17^ caution about the possibility of increased morbidity and mortality in patients with decompensated cirrhosis, although some studies have shown good outcomes for BMS in cirrhotic patients. All guidelines recommend an individualized approach for patients with obesity and metabolic syndrome undergoing BMS.

According to a meta-analysis cited by ABESO, SBH, and SBEM^21^, both vertical sleeve gastrectomy and Roux-en-Y gastric bypass are similarly effective in controlling MASLD^10^. It is worth noting that patients with advanced liver disease may need a liver transplant in the future^7^. Therefore, we consider vertical sleeve gastrectomy the most appropriate technique, as it maintains intestinal transit, removes the gastric fundus (a site with variceal risk), does not leave blind loops, and functions as an azygo-portal disconnection, contributing to reduced portal flow and porto-mesenteric system hypertension.

### MASLD, bariatric surgery, and liver transplantation

MASLD is already one of the leading causes of liver transplantation worldwide^3^. ABESO, SBH, and SBEM^18^ highlight that the presence of liver fibrosis is directly related to disease progression, hepatic decompensation, and mortality or the need for liver transplantation. Any patient with FIB-4>2.67, VCTE>20 kPa, and/or ELF^®^>9.8 should be evaluated by a hepatologist and the transplant team before undergoing BMS. Cases where patients require both BMS and liver transplantation are becoming increasingly common^7^. However, there is still no evidence for the optimal timing of BMS — before, during, or after transplantation. This decision requires further investigation in the coming years. Published studies on this subject are, currently, case reports or case series with small samples^4^. Most articles describe BMS after transplantation, likely because patients are advised to seek bariatric surgery centers for weight loss to avoid graft wear. Chierici et al.^7^ showed in their meta-analysis that simultaneous procedures have low morbidity and mortality, while BMS after transplantation presents increased morbidity. However, performing BMS before liver transplantation is a viable option that can improve the clinical liver function of patients awaiting transplantation. It is important to note that many centers do not perform liver transplantation in patients with a body mass index >30, so BMS before transplantation may be the only chance for these patients.

### Follow-up after biopsy results

Most guidelines emphasize the importance of follow-up and monitoring with a specialist in patients with MASLD, especially those with advanced fibrosis and at risk of cirrhosis and HCC. ABESO, SBH, and SBEM^18^ and EASL^13,16^ suggest performing blood tests and VCTE every three years for patients with F0, F1, and F2 fibrosis and every six months for patients with F3 and F4 fibrosis. NICE^10^ recommends the use of ELF^®^ for regular monitoring and detection of advanced liver disease in high-risk patients. FIB-4 should not be used for follow-up, as age interferes with its absolute value, increasing the number of false positives. Percutaneous biopsy for follow-up after BMS can be considered for those patients with advanced fibrosis at the time of diagnosis or for patients with worsening VCTE and/or ELF^®^ values. After ruling out the possibility of associated HCC, surveillance needs to be continued with a specialist, as suggested by AASLD^19^.

## CONCLUSIONS

Screening for MASLD in patients with obesity and metabolic syndrome in the preoperative phase of BMS should be conducted according to available resources. However, the FIB-4 index is a simple and easily accessible screening tool and should be the first approach in all bariatric patients before surgery; however, it should not be used for patient follow-up. VCTE is a useful tool for a second evaluation after FIB-4, but more studies are needed to better define the cutoff points for the obese population. Family screening and counseling can have a significant impact on the natural history of the disease and should be performed in patients with advanced disease, particularly in the children of relatives with advanced liver disease. Although the ELF^®^ test is highly accurate for screening and monitoring, it is not yet widely available in most centers. BMS should always be considered in the treatment plan for MASLD in patients with a body mass index >35. Liver biopsy remains the gold standard for diagnosis and should be strongly recommended during surgery when there is uncertainty about the stage of fibrosis at screening. Patients with signs of advanced disease need to interrupt the bariatric surgery process and be evaluated by a hepatologist. In cases where liver transplantation is indicated, BMS should not be immediately dismissed but rather discussed to determine the best approach for each patient and each specific center.
